# Nursing care for a patient with post-penile prosthesis implantation infection: a case report

**DOI:** 10.3389/fsurg.2025.1562319

**Published:** 2025-08-13

**Authors:** Yan Gao, Lina Zhou, Lu Wang, Wenjun Meng, Liling Liu, Huiying Chen, Yanmei Wang

**Affiliations:** ^1^ Department of Urology, Gongli Hospital of Shanghai Pudong New Area, Shanghai, China; ^2^ Department of Urology, The 941 Hospital of Joint Logistics Support Force of Chinese People’s Liberation Army, Lanzhou, China

**Keywords:** infection, penile prosthesis implantation, nursing, case report infection, case report

## Abstract

**Background:**

Three-piece expandable penile prosthesis implantation is the ultimate means to treat penile erectile dysfunction, and patients can achieve high levels of satisfaction. However, once postprosthetic infection occurs, clinical treatment and care become very challenging. However, the nursing experience about this disease is rare reported. Therefore, this paper documents the nursing experience of a single case involving postoperative infection following penile prosthesis implantation, with the objective of providing a reference for the clinical management and nursing care of similar cases.

**Case summary:**

We report the case of a 65-year-old male who developed infection after penile prosthesis surgery. By monitoring the pH value, and temperature and by employing topical probiotics, silver ion dressings, light treatment and other methods used to treat infected wounds, we ultimately assisted the physician in choosing the appropriate wound infection plan for the patient. After 60 days of continuous management, the patient's wound healed. Here, we summarize initiatives for the management of such refractory wounds from a nursing perspective.

**Conclusion:**

We summarize the experience in providing nursing care to a patient with a wound infection after penile prosthesis implantation. Our method is very practical and can be applied to similar patients.

##  Introduction

Erectile dysfunction (ED) is a common form of sexual dysfunction in men that not only affects the quality of life of these individuals and their partners but also leads to infertility (1). The implantation of a penile prosthesis is a reliable and effective treatment for ED after oral therapy and physical therapy have failed (2, 3). The frequency of penile prosthesis implantation has steadily increased since the 1970s (4). With improvements in materials and surgical techniques, the patient satisfaction rate after penile prosthesis implantation has reached 80% to 90% (5), and the incidence of postoperative complications has also decreased (4). However, complications such as mechanical failure, infection and erosion can still occur. Penile prosthesis infection is the most serious of these complications and can lead directly to surgical failure, causing pain, local abscess formation and even sepsis, which requires immediate hospitalization and reoperation (6, 7). Once a penile prosthesis infection occurs and is not adequately controlled by antibiotics, the surgeon will typically remove all prosthetic components from the surgical site, resulting in a wound that may be challenging to heal. Nurses play an important role in managing such wounds. However, current literature lacks standardized guidelines for nursing care following penile prosthesis implantation infections.In November 2023, we admitted a patient with infection 10 days after penile prosthesis implantation. After conservative treatment failed, the prosthesis could only be removed. Afterward, the wound was repeatedly infected. Here, we describe the management strategy for this refractory wound after prosthetic infection, emphasizing that in addition to physician treatment measures, nursing management is an important link in the comprehensive treatment of wound healing in such patients.

## Case report

### General information

A 65-year-old patient with ED opted for a three-piece inflatable penile prosthesis implantation at a hospital after failing to respond to drug and physical therapy. Five days post-surgery, the surgical site exhibited swelling and pain. Despite empirical broad-spectrum antibiotic therapy targeting both Gram-positive and Gram-negative organisms, the wound deteriorated, progressing to ulceration and purulent discharge. Consequently, the prosthesis was explanted. Subsequent management involved daily wound disinfection with povidone-iodine solution, surgical debridement, and application of vacuum sealing drainage (VSD). However, the infection persisted, characterized by ongoing purulent secretion. The patient was subsequently admitted to our hospital for further management. He has a 20-year history of type 2 diabetes mellitus managed with oral gliclazide sustained-release tablets; his fasting blood glucose levels were suboptimally controlled, ranging between 7 mmol/L and 20 mmol/L.

### Physical examination

**Figure 1:**
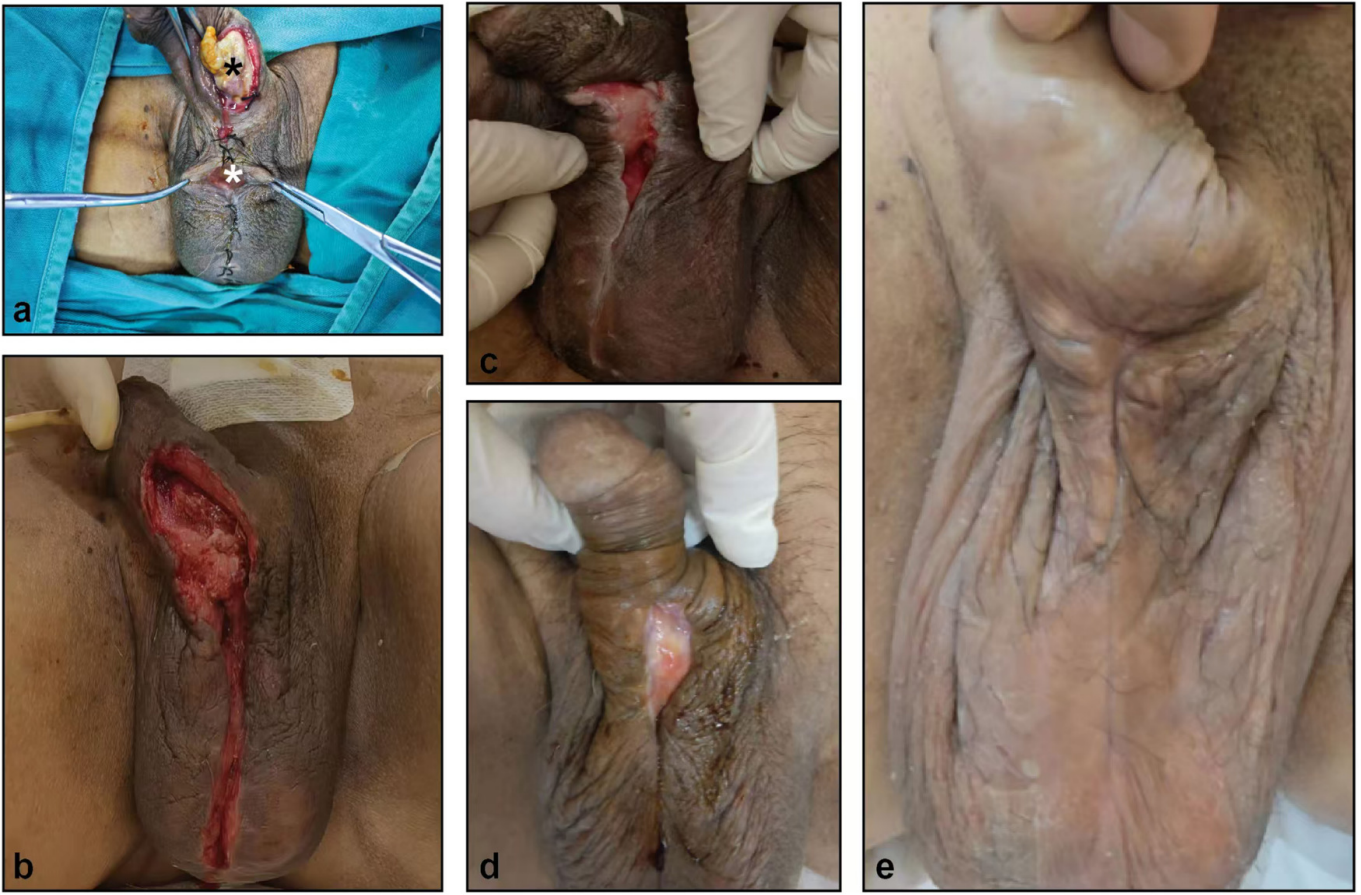
The healing process of penile and scrotal wounds after prosthetic infection. **(a)** penile (black asterisk) and scrotal (white asterisk) wounds. (**b–e)** wounds on day 3 **(b)**, 14 **(c)**, 30 **(d)** and 60 **(e)** after debridement surgery.

There was a wound dehiscence of approximately 5 cm in the penis and a wound dehiscence of 2 cm in the scrotum, accompanied by a large amount of secretion and a distinct odor (Figure 1a). His height was 174 cm, his weight was 75 kg, and his body mass index (BMI) was 24.8 kg/m^2^.

### Laboratory examinations

Routine blood tests revealed a white blood cell count of 15.33 * 109/L, a neutrophil percentage of 79.4%, and a fasting blood glucose level of 8.88 mmol/L. The urinary white blood cell count was 5735.7/µl, and the urinary red blood cell count was 61.4/µl. A plain MRI scan of the genitalia revealed swelling of the scrotal and penile soft tissue, local skin ulceration, and no obvious effusion or pus.

Microbial gene sequencing: Gene sequencing of pathogens present in secretions from the deep wounds of the penis and scrotum revealed the following: bacteria (71.4% *Klebsiella pneumoniae*, 4.7% *Escherichia coli*, 0.5% *Prevotella billoides*, 0.3% *Corynebacterium striata*, 0.1% *Enterococcus faecalis*, 0.1% *Fingolderia magna*), fungi (98.8% *Candida albicans*), and DNA viruses (CMW, circovirus, EBV).

### Treatment

After admission, the physician performed a culture of the secretions and a gene sequence analysis to identify pathogenic microorganisms in the deep wounds of the penis and scrotum; thereafter, sensitive antibiotics were administered to treat the infection according to the results of the urine culture and drug sensitivity tests.Because the patient's blood sugar control was not good, the doctor adjusted the oral hypoglycemic drug regimen to control the fasting blood glucose at about 6–10 mmol/L. After a comprehensive evaluation, the physician performed a resection of the penile lesions + resection and debridement of the penile scrotal skin and subcutaneous tissue, removed all the necrotic tunica albuginea and curetted the granulation tissue, and then the wound was subjected to vacuum-sealing drainage (VSD). After one week, the negative pressure drainage tube was removed, and the dressing was changed regularly. Three weeks after surgery, the wound was significantly improved and the catheter was removed (Figures 1b–e).

### Outcome and follow up

After the removal of the catheter, the patient could urinate by himself. Complete wound healing was achieved after 60 days of treatment and the pain in the perineum disappeared. Follow-up for more than half a year showed no abnormality in urination and wound.

## Main points of nursing care

### Wound surface local care management strategy

#### Wound temperature monitoring

In the process of evaluating wound infection and healing, local monitoring of and intervention into the external microenvironment of the wound are very important. Temperature is an important parameter for determining whether an organism is in a normal state. We used an infrared thermometer to monitor the external body temperature of the patient's wound at 0:00, 3:00, 6:00 and 9:00 and compared this temperature with the body surface temperature of the patient's forehead to evaluate the wound status. Far-infrared light therapy instruments were used to irradiate the wound to maintain the wound temperature at 36–38℃, thus improving the wound microenvironment and promoting wound healing.

#### Therapeutic cleaning

Because the wound had a mixed bacterial infection and most bacterial pathogens often reproduce most rapidly in an alkaline environment, it would have been difficult for these organisms to survive in a neutral or weakly acidic environment. Therefore, when the physician was handling the wound surface, the nurses used a sterile cotton swab to collect the wound secretions and ensured that the glass plane electrode tip of the pH meter fully contacted the wound exudate. After 30 s, two nurses read and recorded the pH value of each wound. On the basis of the pH value of the patient's wound, we chose 0.01% hypochlorous acid and pulsed it with a 20 ml syringe connected to the suction tube until the effluent was clear. If the pH value of the patient's wound was <7 and was in the acidic range conducive to growth, it was not acidified or irrigated with iodine.

#### Use of probiotics

The nurse gave the patient a daily oral supplement consisting of probiotic capsules that included *Lactobacillus fermentum*, *Lactobacillus acidophilus*, *Lactobacillus casei* and *Bifidobacterium bifidum*. Each day, after the wound was debrided and cleaned, the nurse applied probiotics directly to the wound surface until the wound healed.

#### Local anti-infection treatment of the wound

After therapeutic cleaning and debridement, a silver ion dressing was applied locally to the patient's wound to prevent infection. After the silver ions in the dressing came into contact with the wound, they were continuously released into the wound surface, denaturing the bacterial proteins and reducing the number of microorganisms on the wound surface. Microbial smears and cultures of the wound surface were performed every 3 days, and the silver ion dressing application was stopped after the bacterial smear and culture became negative. In this case, silver ion dressings were used for a total of 21 days.

#### Wound light treatment

Far-infrared light treatment (light treatment with an energy density of 0.1–10 J/cm^2^ and a wavelength of 405–1,000 nm) was used to irradiate the samples twice a day, and the irradiation time was 20 min each session. Far-infrared light therapy can promote protein synthesis and energy metabolism, modulate the synthesis and secretion of multiple cytokines, inflammatory mediators and growth factors, relieve pain and interfere with the formation of bacterial biofilms. This patient's wound was treated with far-infrared light for a total of 35 days, and the lamp distance was controlled to avoid thermal damage when the light was being used.

#### Wound protection

In addition, the nurse also used a bed quilt frame to support the quilt and other coverings and thereby protect the skin of the wound and reduce wound friction.

### Systemic wound management strategies

#### Blood glucose management

Capillary blood glucose monitoring was performed daily, and the point-of-care testing (POCT) method performed at the hospital was used to measure the patient's blood glucose levels before each of his three daily meals and before he went to bed. Owing to poor glycemic control, in addition to oral gliclazide extended-release tablets (60 mg qd), 0.5 g tid metformin and 50 mg tid acarbose were also administered orally. The patient's blood glucose changes were monitored daily to control his fasting blood glucose level in the range of 5.6–10.0 mmol/L and his bedtime blood glucose level in the range of 8.3–13.9 mmol/L.

#### Nutritional support treatment

After admission, the 2002 nutritional risk screening scale (NRS2002) was used to evaluate the patient's nutritional status. Due to the patient's wound pain and poor appetite, the overall NRS2002 score was 3 points, and there was a risk of malnutrition. Although the patient's serum albumin concentration was >30 g/L, the nurse supplemented his diet with protein at a daily level of 1.2–1.5g/kg and calories at a daily level of 25–30 kcal/kg to promote wound healing (6).

#### Pain management

A daily visual analog scale (VAS) was used for pain assessment. When the patient was admitted to the hospital, he was in obvious pain, which was rated as 4 on the VAS scale. Therefore, the physician prescribed a 200 mg oral celecoxib capsule to be given 1 h before the dressing was changed every day. In addition, the nurse instructed the patient to use nondrug treatment methods, such as electrophysiologically appropriate techniques (twice a day, 20 min each time, mode: Ble2-002, patch position: patch 5 mm away from the perineal wound, frequency of 35 Hz 320 µs); listening to favorite music; engaging in meditation; use of distraction techniques, etc. The patient's pain was relieved, and satisfactory results were achieved.

#### Catheter management

The patient received an indwelling urinary catheter after implantation of the prosthesis and an indwelling cystostomy tube after debridement. The patient was instructed to drink more than 2,000 ml of water every day to ensure sufficient urine output; observe the color, amount and nature of the urine every day; and record his observations. The urinary catheter and bladder fistula tube were properly fixed, the drainage system was kept unobstructed, and the drainage bag was positioned lower than the urethral orifice or fistula orifice to prevent the catheter from being twisted, compressed or dislodged.

#### Psychological support

Following prosthesis failure due to infection, the patient experienced impaired wound healing and refractory pain, resulting in significant physical and psychological distress manifesting as pessimism and despair. Psychological assessment using the Distress Management Screening Measure (DMSM) (8) revealed moderate distress (score: 5/10) post-diagnosis. The patient initially expressed reluctance regarding condition-related discussions. After evaluating his disease awareness, structured psychological counseling was initiated in a private setting. Clinicians established trust by attentively addressing concerns, creating a supportive environment, and providing comprehensive education on erectile dysfunction, wound infections, and the rationale for diagnostic/therapeutic strategies. Timely feedback on wound progression and dressing efficacy was provided to the patient and family, reinforcing confidence in management. These interventions improved treatment comprehension and cooperation. Notably, the DMSM score declined to 1/10 at discharge.

## Discussion

With improvements in prosthesis quality and surgical skills, the rate of infection after penile prosthesis implantation is decreasing annually; however, once this complication occurs, severe consequences can ensue. Infection after the implantation of a penile prosthesis mostly occurs in the following cases: the patient is diabetic, aseptic procedures are not strictly followed during the operation, the components of the penile prosthesis are in contact with the patient's skin during the operation, or antibiotics are not used correctly (4, 9). In the present case, 10 days after the implantation of the penile prosthesis, there was persistent postoperative pain, fever, swelling of the scrotum, discharge of pus from the penile and scrotal wounds, etc. The physician identified the pathogenic microorganisms according to the wound-culture and gene- sequencing results and applied antibacterial, fungal and viral antibiotics for symptomatic treatment. The excision of necrotic tissue by debridement with VSD negative pressure, combined with subsequent daily dressing changes, promoted wound healing. Although such measures taken by physicians are very helpful in controlling infection, nurses also play a very important role in wound management.In this wound management process, we followed the clinical practice principles of wound infection developed by the International Wound Infection Institute (IWII) (10).

Temperature has long been regarded as an important factor affecting wound healing. When the body produces an immune response, inflammatory cytokines induce vasodilation, and tissue metabolism increases, which causes changes in the temperature of the wound and surrounding skin. 33℃ is a critical temperature for various biological changes in wounds. When the wound temperature is less than 33℃, the activity of neutrophils, Fbs and KCs decreases (11). Power (12) confirmed that improvements in wound conditions are closely related to increases in the wound temperature. When the wound temperature is increased to 36–38℃, the wound area becomes significantly smaller (13). This is because local heating can increase capillary blood perfusion and the partial pressure of blood oxygen; increase neutrophil, Fb and KC activities and collagen deposition; increase the proportion of local lymphocytes in the wound; and enhance the innate immunity of the wound healing microenvironment, thereby improving the wound status and promoting repair. In daily wound management, few physicians pay attention to the temperature of the wound. In the present case, nurses monitored the patient's wound temperature every day. Moreover, to maintain the wound temperature at 36–38℃, we irradiated the wound by using devices emitting far infrared lights. During irradiation, light treatment devices must be kept an appropriate distance from the wound to avoid thermal damage to the skin while improving the microenvironment of the wound and promoting wound healing.

In terms of wound management, physicians perform early debridement, which can not only remove necrotic tissue and infectious secretions but also reduce the number of bacteria, stimulate the production of local growth factors and reduce local pressure. Moreover, when treating wounds, nurses use silver ion antibacterial dressings to effectively control wound infection and improve the wound healing speed, which is consistent with the findings of many studies (14–16). Notably, nurses also pay attention to the pH of the wound. In routine clinical nursing operations, normal saline is often used to clean the wound surface; however, the ability of saline washes to remove bacterial biofilms is very limited. In the context of diabetic foot care, Jeong (17) suggested that a weakly acidic environment can promote a decrease in protease activity and the formation of new blood vessels, strengthen immune mechanisms, inhibit bacterial growth and promote wound healing. However, an alkaline environment not only is favorable for the growth of pathogenic bacteria, but it also inhibits the function of fibroblasts and keratinocytes and delays wound healing (18). Jull (19) applied hypochlorous acid solution to the care of patients with lower extremity venous ulcers and found that the healing time of ulcers was significantly shortened. Therefore, in the present case, the nurse used the nursing management strategy for diabetic foot as a reference. In this patient, hypochlorous acid was used to acidify the ulcer wound, which effectively destroyed the live environment of the pathogenic bacteria and improved the ability to prevent and control infection on the basis of routine wound care to reduce the bioburden. To our knowledge, no studies have reported the management of pH in infected wounds occurring after penile prosthesis implantation; however, we demonstrated that, by improving the pH of such wounds, wound healing can be promoted, and good results can be achieved. In the future care of similar cases, we can pay attention to the change of PH value of infected wounds to further evaluate and adjust the effect of microenvironment PH value adjustment on infected wounds.

Another innovation of this case is that, for the first time, the nurse paid attention to the healing effect of probiotics on infected wounds after penile prosthesis implantation. Traditional methods such as antibiotics and silver sulfadiazine are used to treat skin wound, but the abuse use has many disadvantages, such as chronic wounds and pathogen resistance. Recent studies have shown that probiotics therapy has promising applications in promoting the healing of various skin conditions, including chronic wounds (20), surgical wounds (21, 22), diabetic foot ulcers (23, 24), oral wounds (25), burns (26, 27), and pressure sores (28). Currently, no scholars have attempted to use probiotics in managing infected penile prosthesis wounds. In this case, we used both oral and topical probiotics to treat the patient's infected wound, which resulted in a faster healing rate. This further demonstrates the potential of probiotics in wound management. Nurses can directly apply probiotics to the surface of local wounds. On the one hand, probiotics can directly produce antibacterial substances to inhibit the proliferation of pathogens and form coaggregated complexes with pathogens to prevent the pathogens from adhering to epithelial cells. On the other hand, probiotics can stimulate the body to produce various immune defensive substances, for example, by inducing wound tissue to produce various cytokines by activating T cells; stimulating mast cells, epithelial cells, and adipocytes to produce antimicrobial peptides; and regulating the localization of tight junction proteins *in vitro* and *in vivo* to enhance epithelial barrier function, prevent the invasion of pathogens or eliminate existing pathogenic microorganisms, thereby preventing the formation of biofilms (29). Moreover, nurses can provide patients with oral probiotics for intestinal regulation, which can also promote wound healing. Kotzampassi (30) proposed that intestinal probiotics and their metabolites can form neuroactive molecules that regulate the secretory activity of neuroendocrine cells in the intestinal mucosa, which can improve the systemic health of patients with skin wound healing disorders through the “gut‒brain‒skin axis”. Oral probiotics can regulate the composition or metabolic activity of the intestinal microbiota, further regulate congenital and adaptive immune responses, and thereby promote skin wound healing; oral probiotics can also promote the absorption of nutrients beneficial to skin wound healing by regulating the intestinal microenvironment, especially vitamins, minerals, and cofactors of enzymes involved in tissue repair (30). In conclusion, both oral and topical probiotics are good treatment choices during wound healing.

## Conclusion

Owing to improvements in materials and technology, the rate of infection of penile prostheses has decreased; however, these infections are still a serious complication and challenging to address. Although wound management by physicians plays an important role in wound healing after prosthetic infection, some wound-management measures taken by nurses, including monitoring the wound temperature, using silver ion excipients, maintaining a suitable wound pH value, and administering probiotics, also play significant roles in wound healing. Our care approach is highly practical and can serve as a valuable reference for the clinical management and nursing care of similar cases.

## Data Availability

The original contributions presented in the study are included in the article/Supplementary Material, further inquiries can be directed to the corresponding author/s.
